# Extracorporeal membrane oxygenation therapy in the COVID-19 pandemic

**DOI:** 10.2217/fca-2020-0040

**Published:** 2020-04-17

**Authors:** Nili Schamroth Pravda, Miri Schamroth Pravda, Ran Kornowski, Katia Orvin

**Affiliations:** 1 Department of Cardiology, Rabin Medical Center, 39 Jabotinsky Street, Petach Tikva, Israel 49100; 2 The Faculty of Medicine, Tel Aviv University, Tel Aviv, Israel 6997801; 3 Department of Internal Medicine A, Meir Medical Center, 59 Tchernikovsky Street, Kfar Saba, Israel 4428164

**Keywords:** COVID-19, ECMO, extracorporeal membrane oxygenation therapy

The coronavirus disease 2019 (COVID-19) pandemic is upon us. We are learning and experiencing this viral tsunami with limited published data regarding management guidelines and patient outcomes. COVID-19 can cause severe disease requiring intensive care, extracorporeal membrane oxygenation (ECMO) could be of use in those unresponsive to conventional care. Providing complex therapies such as ECMO during outbreaks of emerging infectious diseases has unique challenges – we aim to review ECMO as a mechanical support option in COVID-19 patients with critical disease.

Severe acute respiratory syndrome coronavirus-2 is the virus that causes COVID-19 disease. The spectrum of disease severity is wide, ranging from asymptomatic patients to those in whom the most serious manifestations of the disease are pneumonia, acute respiratory distress syndrome (ARDS) and an acute cardiomyopathy. Those that develop severe disease are critically ill, requiring intensive care management and often mechanical ventilation. Despite these measures, initial case series have reported a high mortality rate in those with severe disease [1]. ECMO has developed from being used as a ‘rescue therapy’ to become an accepted treatment option for patients with severe ARDS. Providing complex therapies such as ECMO during outbreaks of emerging infectious diseases has unique challenges. We aim to review ECMO as a mechanical support option in COVID-19 patients with critical disease.

## The rational for ECMO

ECMO is a form of mechanical cardiopulmonary support that can provide prolonged extracorporeal life support in patients who are unresponsive to conventional management. There are two forms of ECMO: veno-venous (VV) typically used in cases of severe respiratory failure, and veno-arterial (VA) or veno-venous-arterial (VVA) for patients with cardiogenic shock.

In VV ECMO, blood is extracted from the vena cava or right atrium and returned to the right atrium. This form of ECMO provides respiratory support, but not hemodynamic support, and is used for patients with acute respiratory failure. By replacing the gas exchange function of the lungs, ECMO facilitates protective mechanical ventilation as oxygenation and carbon dioxide clearance are provided by the extracorporeal circuit. This decreases the magnitude of ventilator-induced lung injury secondary to volutrauma, barotrauma and oxygen toxicity.

VA ECMO or VVA ECMO provides both respiratory and hemodynamic support and is used in patients with cardiogenic shock or cardiac arrest. In VA ECMO, blood is extracted from the right atrium and returned to the arterial system, thus bypassing the heart and lungs.

ECMO therapy is associated with potentially severe side effects. These include bleeding and vascular complications, an increased risk of nosocomial infections and poor neurological outcomes. VA ECMO, in particular, provides hemodynamic support but does not reduce the left ventricular afterload and can exacerbated pulmonary congestion.

Some patients exhibit a systemic inflammatory response syndrome due to the exposure of the patient’s blood to the nonendothelialized surface of the ECMO circuit. A potential concern is that the systemic inflammatory response syndrome from ECMO can be detrimental to the recovery process of patients with COVID-19 as lymphopenia and raised IL-6 have been reported to be poor prognostic indicators in these patients. It has been suggested that monitoring the lymphocyte count and IL-6 as prognostic markers could be useful in those with COVID-19 who are treated with ECMO [2].

ECMO therapy is used as rescue therapy in the most critical patients. Therefore, there is a selection bias in that it is used in patients who, from the outset, have significant morbidity and mortality. Patient selection for ECMO should be judicious to optimize patient outcome.

## VV ECMO in ARDS

Reassuring results of the CESAR trial (conventional ventilatory support vs extracorporeal membrane oxygenation for severe adult respiratory failure) [3] and the successful use of ECMO for severe ARDS cases during the influenza A (H1N1) pandemic in 2009 [4] have led to an exponential use of VV ECMO for acute respiratory failure in the last decade. ECMO provides full blood oxygenation and carbon dioxide elimination and allows ‘lung rest’ by enabling low-volume, low-pressure ventilation [5]. It is now considered as a reasonable therapeutic option to support patients with severe acute lung injury refractory to conventional measures. This approach is supported by the Berlin consensus document on ARDS [6].

## VA-ECMO in cardiogenic shock

Short-term mechanical circulatory assist devices such as VA ECMO can be used as a bridging therapy to recovery in those with cardiogenic shock by providing both respiratory and cardiac support, improving end-organ perfusion, reducing filling volumes and augmenting coronary perfusion. However, the outcomes of those on VA-ECMO for refractory cardiogenic shock are poor with an overall reported survival rate of about 40% [7,8] but considerably better prognosis in case of fulminant myocarditis (65–71%) [9]. Prediction tools such as the Survival After Veno-arterial ECMO score have been developed to assist in predicting which patients with cardiogenic shock may have a higher chance of survival with ECMO therapy [10].

## Use of ECMO in previous outbreaks of viral respiratory syndromes

The Extracorporeal Life Support Organisation Registry is an ongoing registry on patients who have been on ECMO support. From these data, 926 adult patients with viral pneumonia of any cause showed an overall survival of 65% with an average duration on ECMO use of 13.5 days [9].

During the 2009 H1N1 influenza A pandemic, almost a third of patients admitted to intensive care units (ICUs) were supported by ECMO therapy [11,12]. Various case series reported a trend toward a decrease in mortality in those who received ECMO therapy [11–13]. An Italian cohort reported a 68% survival to hospital discharge in 61 patients receiving ECMO during the H1N1 influenza A pandemic [11].

Avian influenza A (H7N9) is a viral pneumonia that manifests with varying degrees of dyspnea with most patients developing ARDS and with a subsequent high mortality rate of about 30% [14]. In a review of 35 patients in which ECMO was used for ARDS in patients with Avian influenza A, the ventilator parameters of patients with ECMO, including Fi02 and tidal volume were significantly decreased and physiological parameters, such as pH, were significantly improved. The in-hospital mortality in this cohort was 63% [15].

The Middle East respiratory syndrome (MERS), caused by a coronavirus (MERS-CoV), is characterized by respiratory failure and is associated with a high mortality rate of over 35% [16]. This disease was primarily seen in the Middle East with its’ peak in 2015. In a retrospective analysis on 35 MERS-CoV patients in ICUs with refractory respiratory failure, those who were treated with ECMO had significantly lower in-hospital mortality (65 vs 100%; p = 0.02), compared with those who received conventional therapy. This was also associated with a longer ICU stay (25 vs 8 days; p = 0.001). Of the 21 patients who died, five (14%) died due to uncontrolled hemorrhage and nine (26%) of septic shock [17]. These highlight the potentially fatal complications of ECMO therapy.

## Clinical reports of COVID-19 & the use of ECMO

Reports have recently been published describing the clinical characteristics of this severe disease. A recent report from Wuhan, China, published in *The Lancet* reported that in this cohort six patients (11.5% of COVID-19 cases) in the ICU received ECMO of which only one patient survived [1]. In another report from Wuhan, China, published in *JAMA*, of 36 patients with COVID-19 admitted to the ICU, four patients (11.1%) were treated with ECMO [18]. In a letter in *JAMA*, describing the characteristics and outcomes of 21 critically ill patients with COVID-19 in Washington State, USA, an acute cardiomyopathy was reported in seven patients (33% of the cohort) [19]. It is still unknown if this is due to an acute viral myocarditis or due to overwhelming illness.

The data so far report high rates of potentially fatal complication of COVID-19. However, the clinical courses and outcomes of these patients have not yet been reported and the benefit of ECMO cannot yet be determined.

## Current guidelines in COVID-19 pandemic

The Society of Critical Care Medicine has released tentative guidelines for the management of COVID-19 patients. In these guidelines, they recommend VV ECMO in the management of ARDS in those in whom refractory hypoxemia persist despite optimized ventilation, the use of rescue therapies (such as the use of inhaled pulmonary vasodilators) and proning.

They provide an algorithm to help guide decision-making with the following criteria promoting a recommendation for early ECMO usage:

 paO_2_:Fi02 <80 mmHg for >6 h
 paO_2_:Fi02 <50 mmHg for >3 h
 pH <7.25 with PaCO_2_>/60 mmHg for >6 h

This is currently a weak recommendation with a low-quality evidence [20].

However, this comes with some important caveats. ECMO is a resource-intensive therapy. In the current pandemic and wave of new COVID-19 cases, ECMO should be considered only in those with a good prognosis; those without significant co-morbidities, less than 7 days on mechanical ventilation, and should not be considered in the critically ill elderly due to poor outcomes regardless of ECMO support. Furthermore, ECMO should only be used in resource-stable centers and in settings where personal protective equipment for the staff is not limited.

## Moving forward & ongoing research

In the wake of COVID-19, the Extracorporeal Membrane Oxygenation for 2019 novel Coronavirus Acute Respiratory Disease study is being conducted. Hopefully, this extremely important registry will give us further insights into the role of ECMO in this disease.

*The Lancet* has published a document regarding planning for ECMO preparedness and provision for the COVID-19 pandemic [14]. The high transmission rate of the severe acute respiratory syndrome coronavirus-2 and the ARDS-related mortality are sweeping the world. The number of patients who may need this specialized support is currently unknown. Also unknown is the ability of national healthcare systems to provide this specialized therapy during this resource-straining pandemic.

In conclusion, ECMO is a resource-intensive form of life support that can be used as a rescue therapy in critical patients. ECMO could be of use in COVID-19 patients with severe ARDS or cardiomyopathy in which conventional therapy has failed. The number of patients who might require this level of support is currently unknown and further data are needed.
